# Examining the impact of socioeconomic variables on COVID-19 death rates at the state level

**DOI:** 10.1007/s10818-021-09309-9

**Published:** 2021-03-20

**Authors:** James L. Doti

**Affiliations:** George L. Argyros School of Business & Economics, Gary Anderson Center for Economic Research, Chapman University, One University Drive, Orange, CA 92867 USA

**Keywords:** COVID-19, Empirical, Transmission factors, Public policy, Poverty, C01, C31, C40, C51, I10, I18

## Abstract

This study uses a step-wise regression model to identify the socioeconomic variables most significant in explaining COVID-19 death rates on a state-level basis. The regression tests cover the 1/1/2020 to 12/1/2020 period as well as the first and second halves of 2020. This study also uses the Oxford stringency index to measure more precisely the efficacy of governmental mandates at the state level. The results in this study rigorously showed that while the density variables were the most significant explanatory variables during the first half of the year, their significance fell during the second half. Use of the Oxford stringency index revealed that more stringent mandates led to significant reductions in COVID-19 death rates, especially during the second half of the year. The study’s findings also reveal that a higher poverty rate in a state is significantly associated with higher COVID-19 death rates during all three periods tested.

## Introduction

A number of academic studies have studied the impact of demographic and socioeconomic forces on the incidence of COVID-19. These studies have focused attention on counties and metropolitan statistical areas (Hamidi et al. 2020; Liu et al. 2020; Wheaton and Thompson 2020). At the state level, the print and electronic media have extensively reported on differences in COVID-19 infection and deaths (Olsen, *Washington Post*, 2019; Rosenthal, *New York Times*, 2020; Tavernise and Mervosh, *New York Times*, 2020), but these reports are largely anecdotal and lacking in academic rigor.

The paucity of COVID-19 academic research at the state level is regrettable. State governments have emergency power rules from the Tenth Amendments of the U.S. Constitution to respond to the challenges posed by the COVID-19 pandemic. These rules allow states to enact measures that are not allowed at the local level. As will be presented later in this study, the responsiveness at the state-level in implementing “lockdown style” closure and containment policies will be tested as to their efficacy. These tests are possible because measures of each state’s responsiveness are available at the state level. Such tests are not possible at the city, county, or metropolitan level because uniform measures of local responsiveness are not generally available. Similar data constraints at the local level exist for other socioeconomic variables that might serve in explaining COVID-19 death rates.

Yet another compelling reason for more research at the state-level is that states rather than local areas are increasingly being recognized as laboratories for the control of COVID-19. In a recent Wall Street Journal opinion editorial, for example, a resident fellow of the American Enterprise Institute, Dr. Scott Gottlieb, wrote:

Hospitalizations and deaths are rising, including nursing homes and long-term care facilities. But President-elect Joe Biden can look to some states as a model for handling the pandemic. The good news for Mr. Biden is that he can adopt some of the best practices learned in the states. (Wall Street Journal, 2020)

**Fig. 1:**
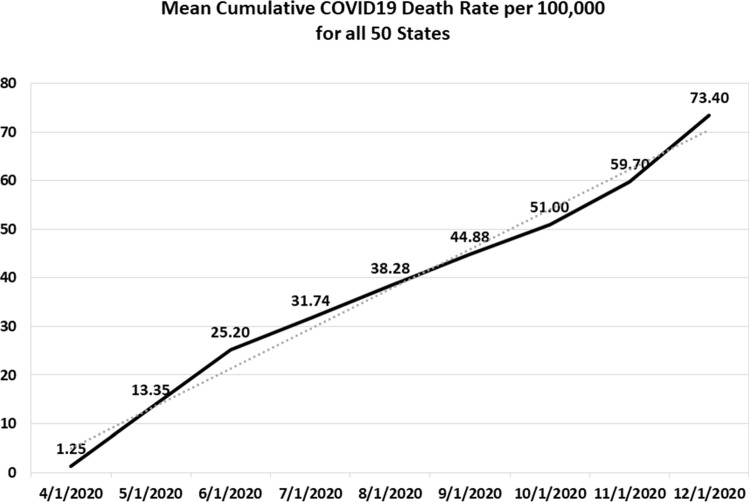
Mean cumulative COVID-19 death rate per 100,000 for all 50 states

State-level COVID-19 death rates vary widely. As shown in the rank ordering of Table 1, cumulative death rates per 100,000 people as of December 1, 2020, range from a low of 10 in Vermont to a high of 191 in New Jersey. The mean cumulative death rate for all 50 states was 73.4, with a standard deviation of 40.5. Figure 1 shows that the mean death rate for all 50 states has increased in a linear-like manner from April 1, 2020, to December 1, 2020.

**Table 1:** As of December 1, 2020

Rank	State	COVID death rates per 100,000 people
1	Vermont	10
2	Maine	14
3	Alaska	17
4	Hawaii	17
5	Oregon	21
6	Utah	27
7	Washington	35
8	Wyoming	37
9	New Hampshire	39
10	West Virginia	41
11	Kentucky	42
12	Oklahoma	44
13	California	48
14	Virginia	48
15	North Carolina	50
16	Idaho	51
17	Nebraska	51
18	Colorado	52
19	Kansas	52
20	Ohio	55
21	Wisconsin	60
22	Missouri	63
23	Montana	63
24	Minnesota	64
25	Tennessee	67
26	Nevada	69
27	Alabama	73
28	New Mexico	73
29	Texas	75
30	Iowa	76
31	Maryland	77
32	Delaware	79
33	Pennsylvania	80
34	Arkansas	82
35	Indiana	84
36	South Carolina	85
37	Florida	86
38	Georgia	89
39	Arizona	91
40	Michigan	95
41	Illinois	102
42	South Dakota	107
43	North Dakota	122
44	Rhode Island	127
45	Mississippi	128
46	Louisiana	138
47	Connecticut	139
48	Massachusetts	156
49	New York	178
50	New Jersey	191
	Average	73.4
	Standard deviation	40.5

Although no state-level studies that examine the impact of socioeconomic variables on COVID-19 have been published, a survey of SSRN as of December 1, 2020, showed 6056 articles dealing with the coronavirus. Of those, 3634 relate to public health, legal, economic, societal, and fiscal implications. Several of these studies focus attention on the impact of density on COVID-19 infection and death rates on counties and metropolitan areas. Hamidi et al. (2020), for example, conclude that their most important finding is “that density is unrelated to confirmed virus infection rates and inversely related to confirmed virus death rates” (p. 11). They conclude that “COVID-19 death rates are lower in dense counties and higher in less dense counties” (p. 12). Wheaton and Thompson’s (2020) findings reveal that density and the total number of infections are inversely related, but that density has no significant effect when the infection rate serves as the dependent variable. These findings run counter to the commonly accepted view that greater social interaction leads to higher COVID-19 infections and deaths. As reported in NYC/EDC:

In theory, population density increases the contact rate of an individual, thereby increasing the production number (also called R by epidemiologists) of the virus and leading to larger outbreaks. (Zhong and Teirlinck, NYC/EDC, 2020)

Determining whether density does or does not play a significant role in explaining the COVID-19 death rate has important implications for socioeconomic planning and policies. As Hamidi et al. (2020) conclude:

The fact that density is unrelated to confirmed virus infection rates and inversely related to confirmed death rates is important, unexpected, and profound. It has important implications for community design, … and nearly every other front-burner issue important to planners. (2020, p. 12)

In the study to follow, we hope to shed light not only on how density and other factors such as state-level mandates are associated with the COVID-19 death rate but also why our findings differ from the conclusions reached in previous studies. We present an empirical model and the results of regression tests to explain these differences in death rates at the state level during three different time periods. The tests regress state-level COVID-19 death rates against hypothesized demographic and socioeconomic explanatory variables. Those variables found to be significant in this study will also shed light on the role these variables play in explaining COVID-19 deaths.

## The model

Cumulative COVID-19 death rates per 100,000 people by state for three different time periods serve as dependent variables in the model. A death is defined as a person dying that tested positive for the coronavirus no matter a person’s preexisting health conditions. COVID-19 virus infection rates were not included in this study because of potential biases due to state-level differences in testing methodologies and people’s varying access to such tests. In addition, antibody tests suggest that coronavirus infections vastly exceed the official counts and that the accuracy of the kits used in the tests is not reliable (Mallapaty, *Nature*, 2020).

The structural form of our model is shown below in Eq. 1, and the functional form of the equation is presented in Eq. 2.

1 $$ {\text{D}}_{{{\text{i}},{\text{t}}}} = {\text{b}}_{0} + {\text{b}}_{1} ({\text{x}}_{{\text{1,i}}} ) \, + {\text{ b}}_{2} ({\text{x}}_{{\text{2,i}}} ) + \cdots + {\text{b}}_{{\text{n}}} \left( {{\text{x}}_{{{\text{n}},{\text{i}}}} } \right), $$

where D_i,t_ is cumulative COVID-19 death rates per 100,000 in state i at the end of some period t. ${\mathrm{x}}_{1}$,…,x_n_ = 1,…,n independent variables in state i. b_0_, b_1_,…,b_n_ = n parameters to be estimated.

Note: display of error terms are suppressed.

Equation (1) can also be estimated in exponential form using natural logs (ln).

In order to control and test for the factors that explain COVID-19 death rates by state, the following demographic and socioeconomic variables shown below in Eq. 2 were selected.

2 $$ \begin{aligned} {\text{Death}}\;{\text{rate}}_{{{\text{i}},{\text{t}}}} & = {\text{b}}_{{\text{o}}} + \sum\limits_{{{\text{d}} = 1}}^{3} {{\text{b}}_{{{\text{d}},{\text{t}}}} } {\text{Density}}_{{\text{i}}} + \sum\limits_{{{\text{y}} = 1}}^{2} {{\text{b}}_{{{\text{y}},{\text{t}}}} } {\text{Income}}_{{\text{i}}} \\ & \quad + \sum\limits_{{{\text{r}} = 1}}^{3} {{\text{b}}_{{{\text{r}},{\text{t}}}} } {\text{Racial}}/{\text{Ethnic}}_{{\text{i}}} + \sum\limits_{{{\text{m}} = 1}}^{3} {{\text{b}}_{{{\text{m}},{\text{t}}}} } {\text{Mandates}}_{{\text{i}}} \\ & \quad + \sum\limits_{{{\text{h}} = 1}}^{4} {{\text{b}}_{{{\text{h}},{\text{t}}}} } + {\text{Health}}_{{\text{i}}} , \\ \end{aligned} $$

where Death rate_i,t_ is cumulative COVID-19 deaths per 100,000 in state i at the end of some period t. b_o_, b_d_, b_y_, b_r_, b_m_, b_h_ are parameters to be estimated.

Note: displays of error terms are suppressed, and the independent variables are as shown in Table 2.

**Table 2:** Dependent and independent variables used in the study

Description	Name	Mean	SD	CV	Min	Max	Obs	Source
Dependent variables								
Death rates from COVID-19 from 1/1/2020 to 12/1/2020	djanjul	34.50	40.54	117.49	10.00	191.00	50	https://www.statista.com/statistics/1109011/coronavirus-covid19-death-rates-us-by-state/
Death rates from COVID-19 from 1/1/2020 to 7/1/2020	djanjul	38.01	38.32	100.83	1.00	169.00		
Death rates from COVID-19 from 7/1/2020 to 12/1/2020	djuldec	41.66	23.28	55.88	1.00	110.00		
Independent variables								
I Density variables								
Population density per square mile	density	202.65	266.24	131.38	1.30	1207.80	50	https://worldpopulationreview.com/state-rankings/state-densities
Super density per square mile	sdensity	342.98	1610.69	469.62	0.00	11,076.00	50	https://en.wikipedia.org/wiki/List_of_United_States_cities_by_population_density
Urban population as a percentage of the total population	urbanpop	0.74	0.15	20.27	0.39	0.95	50	https://en.wikipedia.org/wiki/Urbanization_in_the_United_States
II Income variables								
Per capita personal income (000)	py	54.50	8.80	16.15	39.36	79.09	50	https://fred.stlouisfed.org/release/tables?rid=151&eid=257197
Poverty rate, ratio of persons in poverty	poverty	0.14	0.04	28.57	0.07	0.27	50	https://en.wikipedia.org/wiki/List_of_U.S._states_and_territories_by_poverty_rate
III Racial/ethnic variables								
Black or African American population as a percentage of the total population	afram	10.51	9.55	90.87	0.40	37.60	50	https://worldpopulationreview.com/states/states-by-race
Hispanic population as a percentage of the total population	hispanic	11.74	10.34	88.07	1.50	48.54	50	https://worldpopulationreview.com/state-rankings/hispanic-population-by-state
Asian population as a percentage of the total population	asian	4.18	5.53	132.30	0.76	37.75	50	https://worldpopulationreview.com/state-rankings/asian-population
IV Mandates								
Stringency of state-level mandates from 1/1/2020 to 12/1/2020	sjandec	41.55	7.97	19.18	19.22	59.90	50	https://github.com/OxCGRT/USA-covid-policy
Stringency of state-level mandates from 1/1/2020 to 7/1/2020	sjanjul	36.98	5.92	16.01	22.68	46.69	50	
Stringency of state-level mandates from 7/1/2020 to 12/1/2020	sjuldec	47.00	11.77	25.04	15.10	75.72	50	
V Health related variables								
Percentage of population aged 65 or over	age65	16.49	1.88	11.40	11.10	20.60	50	https://www.prb.org/which-us-states-are-the-oldest/
Obesity rate	obesity	30.75	3.73	12.13	22.60	38.10	50	https://worldpopulationreview.com/state-rankings/obesity-rate-by-state
Diabetes mortality rate	diabetes	21.95	4.39	20.00	14.60	36.20	50	https://www.cdc.gov/nchs/pressroom/sosmap/diabetes_mortality/diabetes.htm
Smoking rate	smoke	17.33	3.50	20.20	8.90	26.00	50	https://worldpopulationreview.com/state-rankings/smoking-rates-by-state

## Empirical findings

A step-wise model was used to add demographic and socioeconomic independent variables to the regression tests arranged in groupings from I to V as shown in Table 2. The results of the regression tests for three different time periods are presented in Tables 3, 4 and 5. In most cases, variables were removed if not significant at the p < 0.10 level (one-tailed). The “best” fit equations in Tables 3, 4 and 5 are shown as shaded in the tables.

**Table 3:**
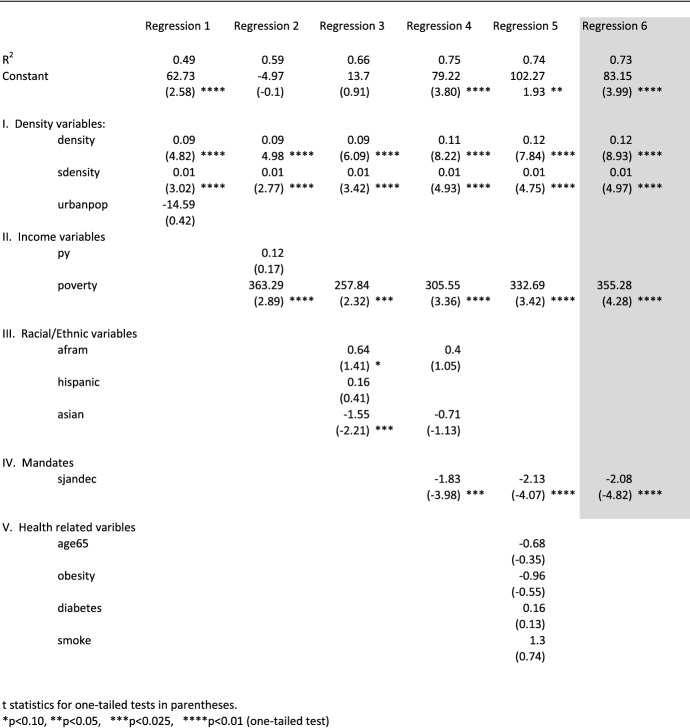
Regression results, dependent variable definition: cumulative death rate (COVID-19 deaths per 100,000 people by state) from 1/1/2020 to 12/1/2020, dependent variable name: djandec

**Table 4:**
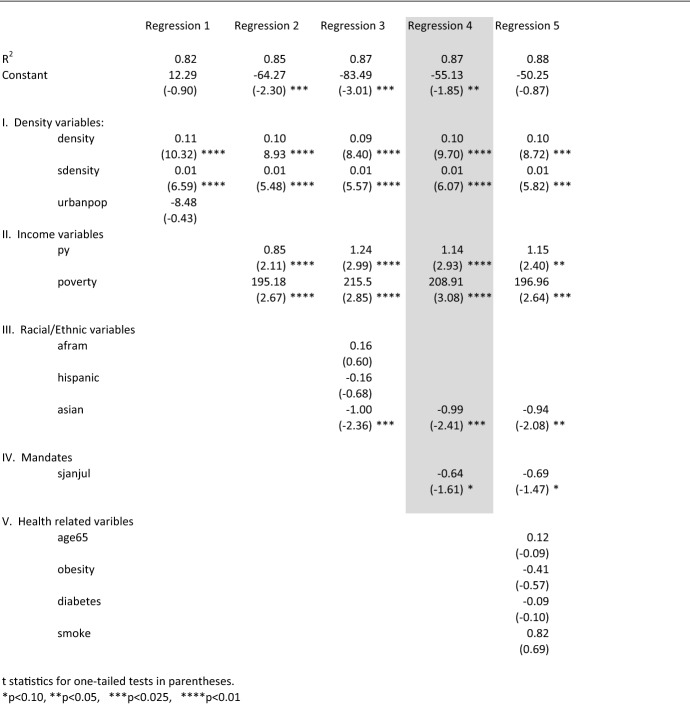
Regression results, dependent variable definition: cumulative death rate (COVID-19 deaths per 100,000 people by state) from 1/1/2020 to 7/1/2020, dependent variable name: djanjul

**Table 5:**
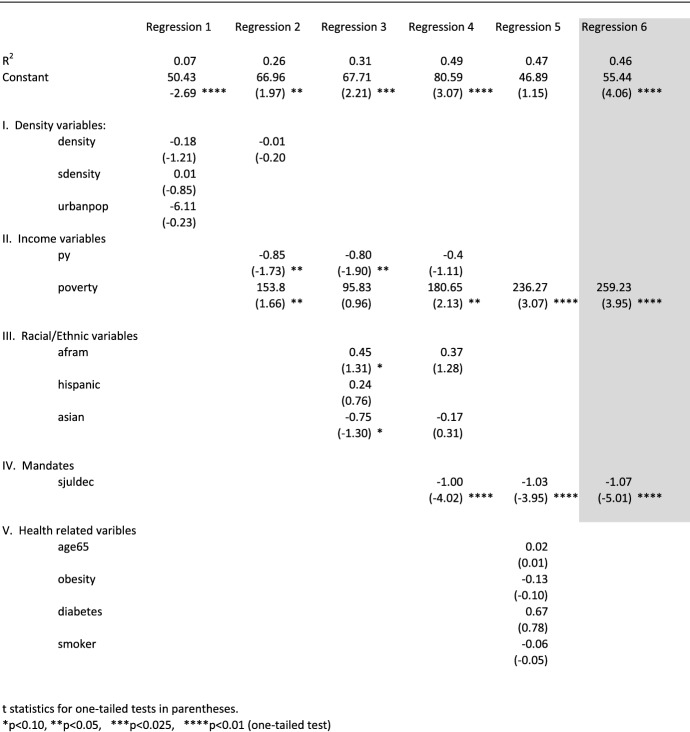
Regression results, dependent variable definition: cumulative death rate (COVID-19 deaths per 100,000 people by state) from 7/1/2020 to 12/1/2020, dependent variable name: djuldec

### Density variables

#### COVID-19 death rates from 1/1/2020 to 12/1/2020

We added a super density variable (sdensity) to our regression tests in the “I. Density variables” grouping because density, as generally measured, does not adequately control for its impact on a state-level basis. A state’s density (density) is defined as the population of that state divided by its total geographic area in square miles or as shown in Table 2: “population density per square mile.” That measure is relevant for most states but not for those states where a highly populated metropolitan area exhibits extremely high density. In those instances, the true nature of a metropolitan area’s density is obscured when dividing by the entire land area of a state. For example, New York City’s density is the ratio of its population of 8.2 million (2010 census) and its land area of 302.6 miles. The resulting density of New York City of 27,016 compares to New York State’s density of 169. Using a state-level density of 169 for New York State would miss the impact of the extraordinarily high rate of density for the city.

In order to capture that impact on a state-level basis, all cities in the nation with a population of 300,000 or more that had a population density of at least 10,000 people per square mile were identified and measured as a ratio of each state’s total population. The resulting ratios, in turn, were multiplied by the density of the metropolitan areas that met the selection criteria presented above.

In the structural form of the model, this density variable (sdensity) is given by

$$ {\text{sdensity}}_{{{\text{i}},{\text{t}}}} = \left[ {\mathop \sum \limits_{{{\text{k}} = 1}}^{{{\text{n}}_{{\text{i}}} }} {\text{p}}_{{{\text{k}},{\text{i}}}} /{\text{P}}_{{{\text{i}},{\text{t}} }} } \right]\;*\;{\text{density}}_{{{\text{i}},{\text{t}}}} , $$

where p_k,i_ is population of the kth city in state i with a population > 300,000 and density > 10,000 per mile^2^. n_i_ is number of cities in state i with population > 300,000 and density > 10,000 per mile^2^. P_i,t_ is population of state i as of some period t. density_i,t_ is density of state i as of some period t.

As shown in Table 3, Regression 1, both density variables (density and sdensity) were highly significant. The urbanization variable (urbanpop) had a negative sign of association and was not significant. The lack of explanatory power for the urbanpop variable is not surprising since urbanization is defined as the proportion of people who live in geographic clusters of 50,000 or more population. No distinction is made in that definition regarding density. Since the spread of COVID-19 is expected to increase when there is close contact, urbanization is too broadly defined to adequately account for virus transmission. The reason for testing its significance is because the print and electronic media continue to use urbanization as a major factor in explaining the spread of the coronavirus (Klaus 2020; Wharton 2020). The regression results in Table 3, Re. 1, suggest its use should be curtailed. Not only was the coefficient for urbanpop insignificant, but when it was removed as an explanatory variable from the regression equation, the R^2^ term remained virtually unchanged at 0.49.

What is particularly noteworthy about the two highly significant density variables is that they explain roughly half of the variation in state-level COVID-19 death rates. Even for statistical outliers like New Jersey and New York, Table 3 Re. 1 shows that density and sdensity explain most of the variation (actual of 191 for New Jersey versus estimated of 159 and actual of 178 for New York versus estimated of 179).

Table 3 indicates that the two density variables (density and sdensity) remained significant as other control variables were added in a step-wise manner. The best-fit equation highlighted in Table 3 suggests that after controlling for other socioeconomic variables that were significant, density and sdensity exhibited the highest degree of explanatory power, as shown by the measured t statistics. This was especially the case with the density variable that had a measured t statistic of 8.93.

The significance of the two density variables described here is in sharp contrast to the results of the studies cited earlier. The reason for these contrasting empirical results is likely related to different methodological approaches as well as the timeliness of the data. In this study, density is measured at the state level and examines COVID-19 deaths through December 1, 2020, while other academic studies focus on the county and/or MSA levels over earlier time periods.

Perhaps a more important factor that accounts for the differences in how density affects COVID-19 is model specification. When Wheaton and Thompson (2020) added population as an explanatory variable to the regression equation that also includes density, the density variable is no longer significant. That does not necessarily mean that density is not a significant factor in explaining COVID-19 infections (cases). More likely, population serves as a proxy for density at the MSA and county levels. As a result, collinearity between population and density may account for the loss of density’s explanatory power. Indeed, the explanatory power of density is robust (p < 0.01) in the Wheaton and Thompson (2020) study when the population variable is not included in their equation.

Hamidi et al. (2020) examined the impact of population and density on COVID-19 infection and deaths at the county level. The regression results suggest that density at the county level is not significant, while the population at the MSA level is significant in explaining infection rates. The density variable is significant in explaining the death rate, but its sign is negative instead of positive, suggesting that higher density decreases rather than increases COVID-19 death rates. The authors suggest that this may be due to “better access to health facilities and easier management of social distancing interventions such as sheltering in place” (Hamidi et al. 2020, p. 12).

More likely, the insignificance of density in explaining infections and the significant negative relationship in explaining death rates in Hamidi, Sabouri, and Ewing’s findings are the result of their model’s construct. In their regression tests of the impact on the rate of COVID-19 infections by county, the density of a county is used as well as the population of the MSA within which the county is located as another explanatory variable. Demographic characteristics of the MSA are likely to be more important in explaining COVID-19 infection and death rates than county-level characteristics. But in their structural equation model (SEM), MSA population likely serves as a proxy for density at the MSA level. Hamidi. Sabouri and Ewing’s conclusion that density is not significant in explaining COVID infection rates may be due to the collinear relationship between population and density at the MSA level. This possibility can be tested in their model by replacing the population variable with density at the MSA level.

This view is supported by regression tests not reported here. When density variables are replaced by population at the state level in Table 3, Re. 6, the population variable is significant but at a lower level than density. In addition, the explanatory power of the equation drops sharply. The lower significance of population as compared to density in Table 3, Re. 4 is not surprising. The states of Maryland and Missouri, for example, have virtually the same population of 6.1 million. But since Missouri is seven times larger than Maryland, its density of 89.3 p/m^2^ is much lower than Maryland’s 622.9 p/m^2^. One would expect that in spite of their equal populations, Maryland is more vulnerable to the coronavirus than Missouri because of its higher relative density. That expected vulnerability will not be captured if population rather than density serves as the explanatory variable.

This problem is also present at the county and MSA levels. If two MSA’s have the same population but different densities, the use of population in place of density as the relevant explanatory variable would suggest that both MSA’s are equally vulnerable to the coronavirus. Given that one of the MSA’s has a higher density, that is not likely to be the case.

The following section will explore how the explanatory power of the density variables varied over two different time periods within the January 1 to December 1, 2020 period.

### Density variables

#### COVID-19 death rates from 1/1/2020 to 7/1/2020 and 7/1/2020 to 12/1/2020

**Fig. 2:**
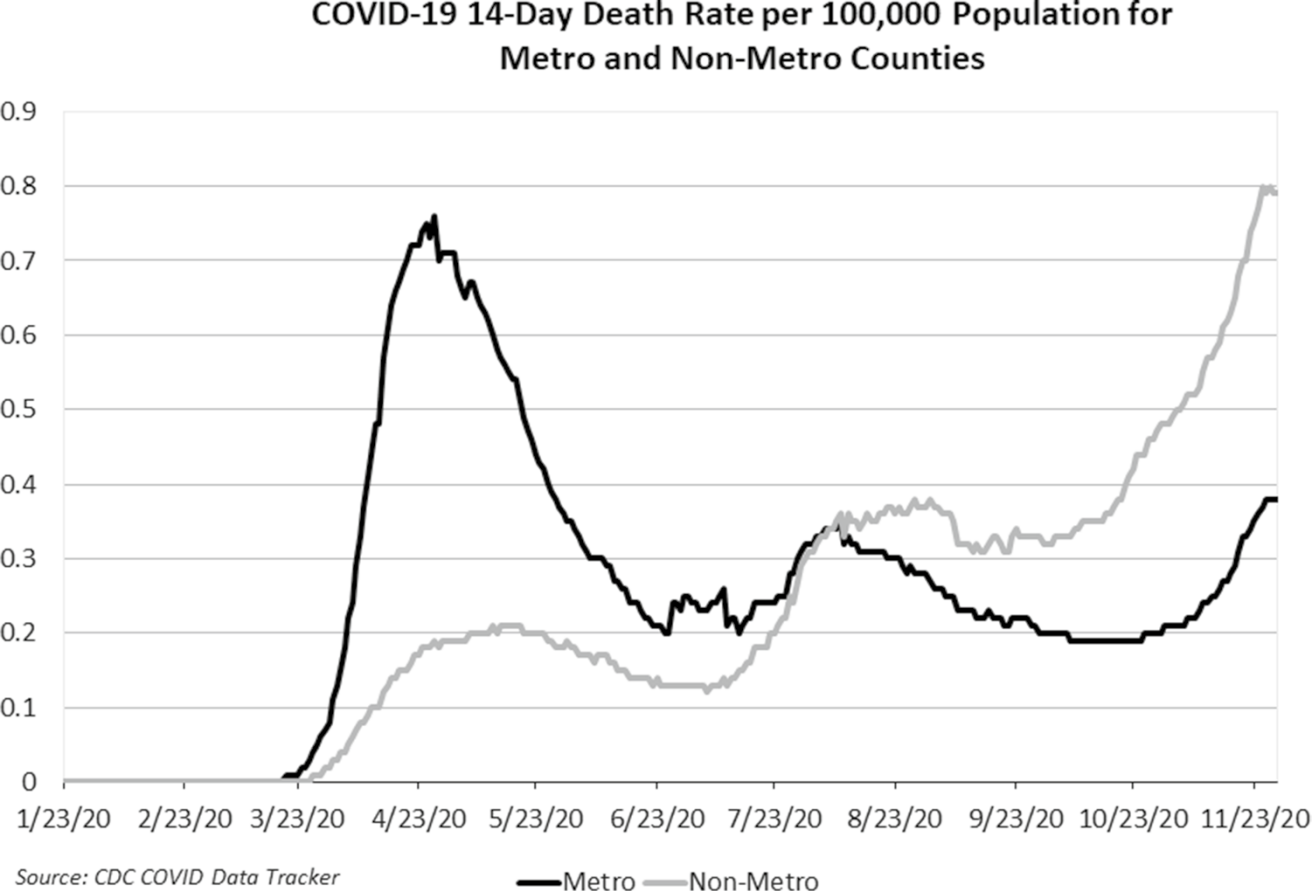
COVID-19 14-day death rate per 100,000 population for metro and non-metro counties

There is some evidence that the impact of density on COVID-19 death rates changed during 2020. As shown in Fig. 2, a 14-day moving average of COVID-19 death rates increased after July 1 for non-metro areas and actually surpassed the death rates for metro areas by August 1.

However, the changing trends shown in Fig. 2 might not be reflected at the state-level since COVID-19 death rates in dense metro areas during the early months of the outbreak may be moving into contiguous non-metro areas in the same state. A better way to determine if there was a change is to examine the relevant correlation coefficient. If the impact of density on COVID-19 deaths did not change significantly at the state level between the first and second halves of the year, the correlation as revealed by Pearson’s correlation coefficient between the average death rates for all states during the two periods would be close to + 1.0. In fact, it was closer to zero and negative at − 0.20. That finding suggests that the impact of density on COVID-19 death rates changed over time. But a more precise way to measure whether the impact of density on COVID-19 deaths at the state level changed during the year is to estimate regression equations over the 1/1/2020 to 7/1/2020 period and then over the 7/1/2020 to 12/1/2020 period. The regression results during those different periods are shown in Tables 4 and 5.

Table 4 shows the regression findings over the 1/1/2020 to 7/1/2020 period. These findings suggest that the explanatory power of both density variables (density and sdensity) were slightly more significant then the impact over the entire January 1 to December 1, 2020 period, as shown in Table 3. The impact of density on COVID-19 deaths, however, changed during the 7/1/2020 to 12/1/2020 period. As shown in Table 4, the two density variables were not significant.

These findings have critically important implications. While COVID-19 hits dense states particularly hard during the first 6 months of the pandemic, all states, on average, appear to be equally vulnerable after 6 months, whether densely populated or not. This calls into question the decisions on the part of those households who relocated to areas less densely populated during the early stages of the pandemic. The findings shown in Tables 4 and 5 suggest that the coronavirus also “relocated” to less densely populated areas.

### Income variables

#### COVID-19 death rates from 1/1/2020 to 12/1/2020

The findings, as shown in Table 3, show that per capita personal income (py) is not significant but that the poverty rate (poverty) is in explaining COVID-19 death rates. The poverty variable was significant and had the expected positive sign of association in each of the step-wise regression tests.

These empirical results suggest that the poverty rate at the state level is a more important variable than personal income in explaining COVID-19 death rates. This is consistent with literature that points to higher poverty rates as increasing the number of confirmed COVID-19 deaths (Finch and Hernandez Finch 2020; Ridgwell 2020).

An example of the powerful influence of poverty in influencing COVID-19 death rates can be observed by comparing two states. The state of Michigan, for example, has the highest poverty rate in the nation at 0.27 while Oregon has the lowest at 0.07. That difference in poverty rates of 0.20 can be multiplied by the regression coefficient of 355.28 for the poverty variable in Table 3, Re. 6 to estimate the impact of poverty on the dependent variable (djandec). The resulting product of 71 (e.g., 355.28 * 0.20) is close to the difference of 74 between Michigan’s COVID-19 death rate of 95 and Oregon’s of 21.

With respect to the per capita income variable (py), the results shown in Table 3 run counter to those studies that point to income as a significant positive or negative factor in explaining the coronavirus. Hamidi, Sabouri, and Ewing’s empirical results, for example, show that counties with a higher percentage of college-educated individuals have significantly lower infection rates. They do not, however, include any variable representing poverty in their tests. Since higher education undoubtedly serves as a proxy for personal income, they may simply be picking up a spurious inverse association between higher education and infection rates because of the collinear relationship between income and poverty that we observed in our empirical findings. Indeed, the Pearson correlation between poverty and personal income is − 0.50 (p < 0.01).

Unlike Hamidi, Sabouri, and Ewing’s findings of an inverse relationship between the percentage of college-educated and COVID-19 infections, Wheaton and Thompson found a significant positive relationship between per capita income and coronavirus cases at the county and MSA levels. The authors were surprised by this result and state, “It is tempting to suggest that perhaps dining out, entertaining, and socialization are all income elastic consumption items—items that also generate higher infection risk. But we need further direct research before drawing that conclusion” (Wheaton and Thompson 2020, p. 9).

Alternatively, our findings suggest that the collinear relationship between income and poverty should be taken into account in order to more accurately assess the impact of personal income.

### Income variables

#### COVID-19 death rates from 1/1/2020 to 7/1/2020 and 7/1/2020 to 12/1/2020

The positive association between poverty and COVID-19 death rates that exhibited a high degree of significance at the one-tailed 0.01 level for the full year regression results shown in Table 3 continued as significant during both periods tested in Tables 4 and 5.

Unlike the full-year results reported in Table 3, the personal income variable showed a statistically significant positive impact during the January 1 to July 1, 2020 period. The fact that the full-year results reported in Table 3 showed no significance for the personal income variable is likely because the positive impact for personal income during the 1/1/2020 to 7/1/2020 period disappeared during the 7/1/2020 to 12/1/2020 period (see Table 5). But the positive association between personal income and COVID-19 death rates during the first 6 months of the pandemic seems anomalous. One would expect a higher average personal income level would allow for more access to health care and a greater ability to work remotely.

The fact that it does not, at least during the first 6 months of the pandemic, may be the result of the effects of agglomeration economics in urban areas. Spatial concentration of economic activities in urban produce areas leads to scale economies and higher value-added jobs. The resulting agglomeration economies in more densely populated areas lead, in turn, to higher personal income levels (Boston MA. O’Sullivan 2008, *Urban Economics*, *6th Edition*, McGraw-Hill College). If personal income, therefore, is related to density, the positive impact of the personal income variable on COVID-19 deaths during the first 6 months of the pandemic may not be because of income effects but as a result of the positive correlation between density and personal income. Indeed, the correlation between py and density is a significant 0.57. Since density was only significant during the January 1 to July 1, 2020 period, it likely explains why the personal income variable was significant only during that first half of the year.

### Racial/ethnic variables

#### COVID-19 death rates from 1/1/2020 to 12/1/2020

Most of the reported findings on the relationships between racial (ethnic) variables point to higher infection and death rates for African-Americans and Hispanics (APM Research 2020; Magnier 2020), but the findings on Asians are mixed. Several studies point to higher rates of COVID-19 infections and fatalities (Health Affairs 2020; McKinsey & Company 2020), while others (APM Research 2020; Magnier 2020) point to significantly lower rates. These studies, however, do not control for the causal relationships of other socioeconomic variables like density and poverty.

The empirical findings in Table 3 show no significant relationship for racial/ethnic variables during the January 1 to December 1, 2020 period after controlling for other socioeconomic variables. Although the Asian-American variable (asian) exhibited one-tailed significance at the 0.025 level in Table 3, Re. 3, it dropped from significance after adding a mandate variable.

It should be noted that in testing the impact of racial/ethnic variables on COVID-19 death rates, we excluded a White racial category because adding it would bring the regression equations we tested close to a singular matrix.

### Racial/ethnic variables

#### COVID-19 death rates from 1/1/2020 to 12/1/2020 and 7/1/2020 to 12/1/2020

As shown in Tables 4 and 5, the only significant racial/ethnic estimated coefficient was that represented by Asian Americans (asian) but only during the 1/1/2020 to 7/1/2020 period. The coefficient of -0.99 for the asian variable shown in Table 4, Re. 4 suggests that if the percentage of Asians in a particular state increases by one, that state’s COVID-19 death rate per 100,000 would decrease by almost one. One might question why there was a significant negative explanatory relationship between the asian variable and the COVID-19 death rate during the 1/1/2020 to 7/1/2020 period but not during the 7/1/2020 to 12/1/2020 period. A possible explanation for this difference is anecdotal evidence that Asian-Americans responded more quickly in adopting safe-distancing and mask-wearing before such preventive measures were mandated by governments to the general population. As observed by Scott Frank, a public health expert at Case Western Reserve University’s medical school:

Mask wearing was something done by Asians well before the beginning of this pandemic. There’s recognition that individual concerns should be subsumed for the good of the whole, rather than the more individualistic ethic that is oriented towards freedom and choice. (https://www.scmp.com/news/china/article/3084947/asians-us-least-likely-get-coronavirus-infection-data-suggests).

### Mandates

#### COVID-19 death rates from 1/1/2020 to 12/1/2020

A great deal of controversy has arisen over the efficacy of governmental mandates that imposed various restrictions in order to control the spread of COVID-19. An article in the *New York Times* (Erdbrink, *New York Times*, 2020) suggests that Sweden’s COVID-19 caseload provides some support for its relatively lax approach in responding to the coronavirus. Others argue that lower cumulative infections and death rates in neighboring Denmark and Norway, two nations that responded more aggressively with government mandates, support greater use of publicly imposed restrictions (*Boston Review*, 2020; Healthline 2020). Even Sweden now appears to be reversing its course by implementing more stringent restrictions as its caseload increased in recent months (*Wall Street Journal*, 2020).

A recent study of mine that attempted to measure the efficacy of mandates found no significant relationship between mandates and COVID-19 death rates at the state-level (Doti 2020, *A Model to Explain Statewide Differences in COVID-19 Death Rates*, SSRN# 3731803). That finding was based on testing the impact of the number of days from March 12 to September 1 before state-level mandates were imposed on wearing masks; the cumulative number of mandates imposed within a 30-day period following March 12, 2020; and a social distance index. In addition to these three variables, five other mandates were aggregated using z values to normalize the data to become a comprehensive “mandate score.”

A major weakness in using this measure to assess the efficacy of governmental restrictions is that most of the mandates that comprise the final score only included measures relating to how quickly they were imposed within a 30-day period following March 12. The other mandates that comprised the aggregate mandate score at most ran through September 1.

In the present study, the “mandate score” described above was replaced by the Oxford daily government stringency index. This index more accurately measures government mandates on a daily basis, using a scale from 1 to 100. The ordinal measures that comprise the Oxford index for every state include in its measurement the following 11 government responses to COVID-19:

- School closings
- Workplace closings
- Cancellation of public event
- Restrictions on gathering size
- Closures of public transit
- Stay at home requirements
- Restrictions on internal movements
- Restrictions on international travel
- Public information campaign
- Testing policy
- Contact tracing

The daily Oxford stringency index used in this study was derived by calculating an average stringency index from the daily rates for each state during the 1/1/2020 to 12/1/2020; 1/1/2020 to 7/1/2020; and the 7/1/2020 to 12/1/2020 periods. The derivation is given by:

$$ {\text{s}}_{{{\text{i}},{\text{t}}}} = \mathop \sum \limits_{{{\text{d}} = 1}}^{{{\text{n}}_{{\text{t}}} }} {\text{s}}_{{{\text{i}},{\text{d}}}} /{\text{n}}_{{\text{t}}} , $$

where s_i,t_ is mean stringency index in state i as of some period t, s_i,d_ is stringency index in state i as of a particular day, d, n_t_ is number of days during period t.

In a study that used a Bayesian model to investigate the socioeconomic factors that explain nationwide differences in the spread of COVID-19 (Stojkowski et al. 2020), the authors used the Oxford stringency index but added inverse weights to give larger weights to earlier dates. Their argument for using such a weighted index was “because earlier restrictions have obviously a bigger impact on the prevention of the spread of the disease” (Stojkowski, et al. 2020, p. 22).

In fact, the empirical results in this study show quite the opposite: the impact of earlier governmental restrictions have a lower impact. As a result, the Oxford index was not weighted in the equations tested in this study.

**Fig. 3:**
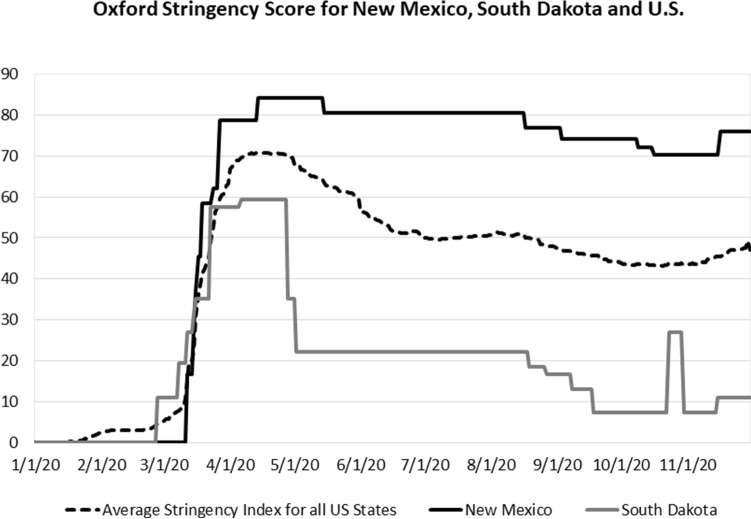
Oxford stringency score for New Mexico, South Dakota and U.S

Figure 3 shows the daily Oxford stringency index for the U.S., and for comparison, it shows the most stringent state (New Mexico) and the least stringent state (South Dakota) over the 1/1/2020 to 12/1/2020 period.

Table 6 shows the average stringency measures calculated for the three-time periods in this study for New Mexico, South Dakota, and the U.S.

**Table 6:** Average values for Oxford stringency index for New Mexico, South Dakota, and the U.S

	1/1/2020 to 12/1/2020	1/1/2020 to 7/1/2020	7/1/2020 to 12/1/2020
New Mexico	59.90	46.69	75.72
South Dakota	19.22	22.68	15.10
U.S. average	41.55	36.98	47.00

The average Oxford stringency index values for all states in rank order from highest to lowest over the 1/1/2020 to 12/1/2020 period are shown in Table 7.

**Table 7:** Average Oxford stringency index values from 1/1/2020 to 12/1/2020 in rank order

Rank	State	Stringency index
1	New Mexico	59.90
2	New York	57.50
3	Hawaii	56.88
4	Maine	55.25
5	Rhode Island	53.84
6	California	50.25
7	Connecticut	49.42
8	Kentucky	48.20
9	Delaware	48.17
10	Vermont	47.55
11	Maryland	47.53
12	Ohio	47.38
13	Massachusetts	46.17
14	North Carolina	45.99
15	Colorado	45.28
16	Minnesota	44.84
17	Illinois	44.33
18	Washington	43.57
19	Virginia	43.47
20	West Virginia	43.31
21	Texas	42.22
22	Florida	41.99
23	Oregon	41.93
24	Louisiana	41.60
25	New Jersey	41.41
26	Pennsylvania	41.31
27	Michigan	41.08
28	Montana	40.66
29	New Hampshire	39.64
30	Alaska	39.58
31	Georgia	39.35
32	Wyoming	39.32
33	Idaho	38.41
34	Tennessee	38.18
35	Nevada	38.12
36	Kansas	37.65
37	Indiana	37.16
38	Mississippi	36.23
39	Missouri	35.99
40	Wisconsin	35.86
41	Nebraska	35.80
42	Arizona	35.34
43	South Carolina	33.68
44	Arkansas	33.47
45	Utah	32.21
46	Oklahoma	29.73
47	Alabama	28.74
48	North Dakota	28.07
49	Iowa	25.92
50	South Dakota	19.22
	Average	41.37

Unlike the empirical results of my previous study (Doti 2020, *A Model to Explain Statewide Differences in COVID-19 Death Rates*, SSRN# 3731803), the regression tests shown in Table 3, Regression 6, point to a highly significant inverse relationship between mandates as measured by the Oxford stringency index and the COVID-19 death rate for all 50 states over the 1/1/2020 to 12/1/2020 period. The measured t statistic of − 4.82 for the stringency index (sjandec) is highly significant at p < 0.01. Its estimated coefficient of − 2.08 suggests that a state’s COVID-19 death rate decreases by 2.08 deaths per 100,000 for every increase of 1 point in the Oxford stringency index.

**Fig. 4:**
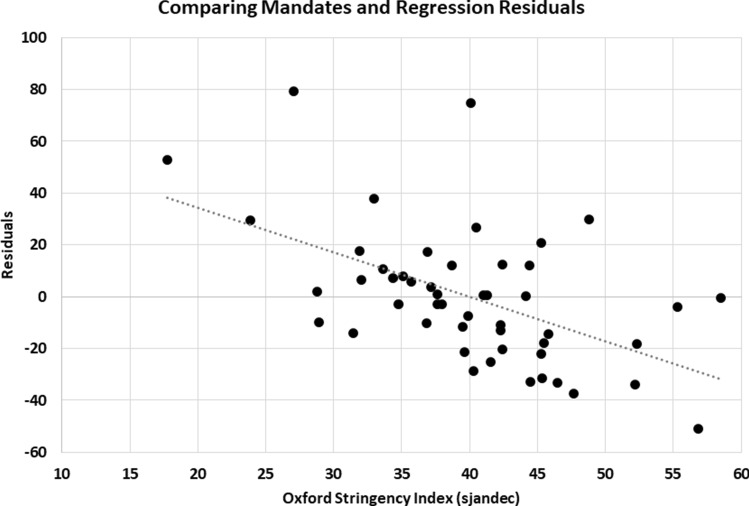
Comparing mandates and regression residuals

Another way of examining the explanatory power of mandates in reducing state-level COVID-19 death rates is to compare R^2^ terms. In an equation (not reported here), the R^2^ term for Table 3, Re. 6, when sjandec is excluded as an explanatory variable, is 0.60. A scatter diagram that compares the residuals from that regression equation with the Oxford stringency index (sjandec) is shown in Fig. 4. The trendline points to the significant explanatory power of governmental mandates. Given these empirical results, it should not be surprising that when sjandec was added back to the equation, the R^2^ term increased from 0.60 to 0.73 (Table 3, Re. 1).

### Mandates

#### COVID-19 death rates from 1/1/2020 to 7/1/2020 and 7/1/2020 to 12/1/2020

The estimated coefficients for the mandate variables, sjanjul and sjuldec, were significant in both the 1/1/2020 to 7/1/2020 period (Table 4) and the 7/1/2020 to 12/1/2020 period (Table 5). But the mandates as measured by the Oxford stringency index were more effective in reducing COVID-19 death rates during the second half of the year.

In the best-fit equation for 1/1/2020 to 7/1/2020 (Table 4, Re. 4), the estimated coefficient for sjanjul was − 0.64. That compares to − 1.07 for sjuldec during the 7/1/2020 to 12/1/2020 period (Table 5, Re. 6). Its measured t statistic of − 5.01 compares to a lower but still marginally significant − 1.61 during the first half of the year.

**Fig. 5:**
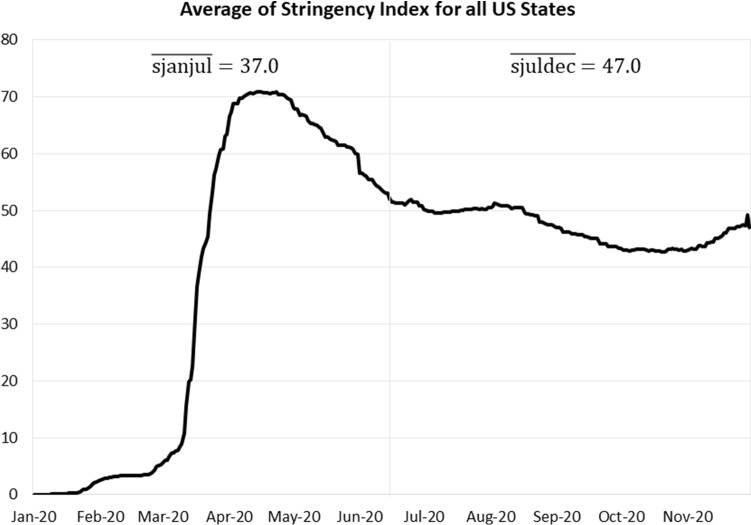
Average of stringency index for all US states

These results suggest that the efficacy of governmental mandates in reducing the COVID-19 death rate, as measured by the Oxford stringency index, increased over time. There are several possible reasons for this. As shown in Fig. 5, it took three months before the average stringency index began increasing. Then after a rapid increase and peaking at 70.82 in mid-April, the index declined through July 1. Thereafter, it leveled off before increasing again in late November with the onset of a surge of the coronavirus.

On average, the stringency index during the 1/1/2020 to 7/1/2020 period (sjanjul) was 37.0 as compared to a higher 47.0 during the 7/1/2020 to 12/1/2020 period (sjuldec).

In addition to states, on average, being less stringent during the first half of the year, it is also likely that governmental responses were less effective when COVID-19 was most virulent, particularly in densely populated states. Recall that the findings in the study show that the density variables were only significant during the 1/1/2020 to 7/1/2020 period. The rapid growth of the coronavirus during its early stage when higher density was fueling a rapid spread of the disease was likely occurring too rapidly to be contained by stringency measures that were late in coming. In addition, during its early stages, an unsuspecting public unfamiliar with the virus may have led many to resist governmental mandates and be more lax in self-administering protective measures.

By July, however, as reflected by the Oxford stringency index, strong governmental measures were in place. A more informed public was also more likely to adhere to these measures. As a result, the effectiveness of governmental mandates increased during the course of the year, as reflected by the higher value for the estimated coefficient and greater significance of the stringency index during the second half of the year, as shown in Table 5.

The estimated coefficients for the stringency variables sjanjul and sjuldec can also be used to estimate the lives saved by a state having a stringency index above the mean index and those lost by having an index below the average. Those estimates are presented in Table 8 and are based on the following derivation:

$$ \Delta {\text{D}}_{{{\text{i}},{\text{t}}}} = [{\text{s}}_{{{\text{i}},{\text{t}}}} - \overline{s}_{{{\text{i}},{\text{t}}}}] *\hat{b}_{{{\text{m}},{\text{t}}}} *[{\text{P}}_{{{\text{i}},{\text{t}}}} /100,000,] $$

**Table 8:** Change in deaths for those states having a stringency index above (+) or below (−) the U.S. average

State	Change in deaths from 1/1/2020 to 7/1/2020	Change in deaths from 7/1/2020 to 12/1/2020	Total change in deaths for both periods
Alabama	364	735	1099
Alaska	10	11	22
Arizona	289	465	754
Arkansas	134	295	429
California	− 1622	− 5071	− 6694
Colorado	− 137	− 259	− 396
Connecticut	− 109	− 451	− 561
Delaware	− 59	− 35	− 95
Florida	− 723	1160	437
Georgia	216	56	271
Hawaii	− 33	− 451	− 484
Idaho	22	83	105
Illinois	− 328	− 204	− 533
Indiana	69	529	597
Iowa	192	769	961
Kansas	37	180	218
Kentucky	− 244	− 262	− 506
Louisiana	102	− 268	− 166
Maine	− 81	− 274	− 355
Maryland	− 350	− 172	− 522
Massachusetts	− 24	− 746	− 771
Michigan	− 67	217	150
Minnesota	− 141	− 170	− 310
Mississippi	96	167	262
Missouri	117	552	668
Montana	− 8	36	28
Nebraska	24	206	231
Nevada	25	190	215
New Hampshire	− 30	118	87
New Jersey	57	− 111	− 55
New Mexico	− 129	− 653	− 782
New York	− 1382	− 4570	− 5952
North Carolina	− 56	− 1007	− 1063
North Dakota	56	126	182
Ohio	− 326	− 990	− 1316
Oklahoma	177	733	910
Oregon	37	− 153	− 116
Pennsylvania	− 51	140	89
Rhode Island	− 62	− 184	− 246
South Carolina	98	741	839
South Dakota	82	298	380
Tennessee	131	192	322
Texas	356	− 1293	− 937
Utah	183	327	510
Vermont	− 16	− 60	− 76
Virginia	− 20	− 365	− 385
Washington	21	− 425	− 404
West Virginia	− 23	− 37	− 60
Wisconsin	123	515	638
Wyoming	5	19	24

where D_i,t_ is change in the number of COVID-19 deaths in state i during some period t, s_i,t_ is the average stringency index for state i during period t, $\overline{\mathrm{s}}$_i,t_ is the mean stringency index for all states during period t, $\widehat{\mathrm{b}}$_m,t_ is the estimated coefficient for the mandate variable during period t, ${\mathrm{P}}_{\mathrm{i},\mathrm{t}}$ is the population of state i at some period t.

### Health-related variables

None of the four health-related variables added to our equation were significant during any of the three periods. As shown in Tables 3, 4 and 5, all measured t’s were below one.

The high degree of collinearity between obesity, diabetes, and smokers is reflected by Pearson correlation coefficients that range between 0.67 and 0.78. Because of this high degree of correlation, we tested regression equations that added obesity, diabetes, and smoking rates individually as separate explanatory variables. Even in these equations (findings not reported here), the coefficients for each of the individual health-related variables showed no significance.

What is most surprising in these results is the lack of significant explanatory power for the variable representing the percentage of the population over 65. Tests were also conducted using the percentage of the population over 80 (not reported here) and obtained similar results that showed no significance between age and death rates.

As shown in Table 9, 80% of all COVID-19 deaths occurred in age cohorts of 65 and above. With COVID-19 death rates disproportionally affecting those in older cohorts, one would expect that the age 65 variable would exhibit a significant positive relationship. The fact that our empirical results reveal no significance seems anomalous, especially in light of findings in the Hamidi et al. (2020) study. Unlike our findings, their SEM tests for the impact of the percentage of population aged 60+ on both the virus rate and the death rates resulted in highly significant coefficients (p < 0.0001).

**Table 9:** Deaths associated with COVID-19 by age group in the U.S. (December 9, 2020)

Age group	No. of deaths	Percent of all deaths	Death rate per 100,000 people in age cohort
All ages	2,61,530	100	79.68
Under 1 year	29	0.01	0.77
1–4 years	17	0.01	0.11
5–14 years	46	0.02	0.11
15–24 years	449	0.17	1.05
25–34 years	1909	0.73	4.16
35–44 years	4917	1.88	11.8
45–54 years	13,080	5	32
55–64 years	31,973	12.23	75.32
65–74 years	55,985	21.41	177.82
75–84 years	70,815	27.08	443.43
85 years and over	82,310	31.47	1246.19

*Source* https://www.cdc.gov/nchs/nvss/vsrr/covid_weekly/index.htm

**Fig. 6:**
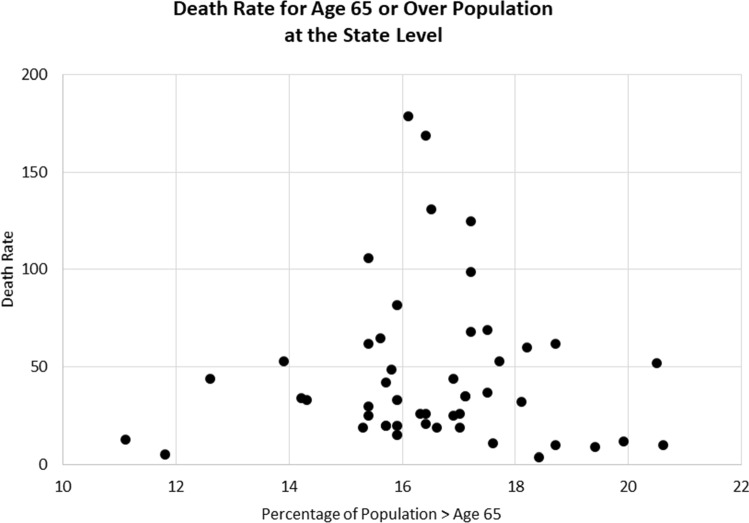
Death rate for aged 65 or over population at the state level

Closer examination of the data, however, offers an explanation for the differing findings. The scatter diagram in Fig. 6 shows that the death rates at the state level occurred with mean state-level ages concentrated near the national average rather than at outlying values.

These findings suggest there is not enough age dispersion in the state-level data for the regression equation to pick up any significant explanatory power. At the county and metropolitan levels, however, the dispersion is greater, as reflected by a coefficient of variation (cv) of 21.6 in the Hamidi et al. (2020) study for their age 60+ variable. The cv for our 65+ variable (age65) at the state level is a lower 11.4. That cv of 11.4, as shown in Table 2, is the lowest cv value for any of the variables we tested.

These results suggest that while age is clearly a significant factor in explaining county and metropolitan COVID-19 death rates, there is not enough age dispersion to accurately measure its impact in regression tests at the state level.

### Elasticities

The elasticities calculated from a linear regression equation are not constant but vary with respect to the point at which the relevant elasticity is being calculated. Since the equations estimated in this study are in linear form, it will be necessary to calculate average elasticities. The derivation for the average elasticity of the COVID-19 death rate with respect to density is presented below.

$$ \overline{{{\text{E}}_{{{\text{i}},{\text{t}}}} }} = \frac{{\partial {\text{D}}_{{{\text{i}},{\text{t}}}} { }}}{{\partial {\text{density}}_{{{\text{i}},{\text{t}}}} { }}}*\frac{{\overline{{{\text{density}}_{{{\text{i}},{\text{t}}}} }} }}{{\overline{{{\text{D}}_{{{\text{i}},{\text{t}}}} }} { }}}, $$

where $\overline{{\mathrm{E}}_{\mathrm{i},\mathrm{t}}}$ is average elasticity of the death rate in state i at the end of some period t, D_i,t_ is death rate per 100,000 in state i at the end of some period t, density_i,t_ is density in state i at time period t, $\overline{\mathrm{density}}$_i,t_ is average density, $\overline{\mathrm{D}}$_i,t_ is average death rate.

In the structural form of our model, this can be derived by

$$ \overline{{{\text{E}}_{{{\text{i}},{\text{t}}}} }} = {\text{b}}_{{\text{d}}} \frac{{\overline{{{\text{density}}_{{{\text{i}},{\text{t}}}} }} }}{{\overline{{{\text{D}}_{{{\text{i}},{\text{t}}}} }} { }}}, $$

where b_d_ is the estimated coefficient for density.

When the functional form of the structural equation is in exponential form such as

$$ {\text{D}}_{{{\text{i}},{\text{t}}}} = {\text{b}}_{0} ({\text{density}}_{{{\text{i}},{\text{t}}}} )^{{{\text{b}}_{{\text{d}}} }} . $$

It can be shown that the constant elasticity, E_i,t_ can be expressed in the double logarithmic form (Doti and Adibi 2019, p. 385) as

$$ {\text{E}}_{{{\text{i}},{\text{t}}}} = \frac{{\partial {\text{D}}_{{{\text{i}},{\text{t}}}} }}{{\partial {\text{density}}_{{{\text{i}},{\text{t}}}} }}*\frac{{{\text{density}}_{{{\text{i}},{\text{t}}}} }}{{{\text{D}}_{{{\text{i}},{\text{t}}}} }} = \frac{{{\text{b}}_{1} {\text{b}}_{0} ({\text{density}}_{{{\text{i}},{\text{t}}}} )^{{{\text{b}}_{{\text{d}}} - 1}} }}{{{\text{D}}_{{{\text{i}},{\text{t}}}} }} = {\text{b}}_{{\text{d}}} . $$

Since the elasticities are most relevant for the 1/1/2020 to 7/1/2020 and 7/1/2020 to 12/1/2020 periods, the calculated average elasticities, $\overline{\mathrm{E}}$, and constant elasticities, $\overline{\mathrm{E}}$ are based on Table 4, Regression 4 and Table 5, Regression 6.

The double logarithmic form of the regression equation upon which the constant elasticities shown in Table 10 are based has an R^2^ value of 0.64 versus the higher R^2^ value of 0.87 for the linear form of the equation (Table 4, Regression 4) during the 1/1/2020 to 7/1/2020 period. Similarly, the R^2^ value for the double logarithmic form of the equation during the 7/1/2020 to 12/1/2020 period of 0.4 was lower than the 0.46 for the linear form of the equation (Table 5, Regression 5). In spite of the lower explanatory power of the double ln form of the equation, elasticities based on those equations have the advantage of being constant across different values of the independent variables. Their drawback is that the estimated coefficients are not as reliable, given the lower explanatory power of the regression equation in double ln form.

**Table 10:** ...

Elasticities during the 1/1/2020 to 7/1/2020 period			Elasticities during the 7/1/2020 to 12/1/2020 period		
Independent variable	Average elasticity	Constant elasticity	Independent variable	Average elasticity	Constant elasticity
	$\bar{E}$	E		$\bar{E}$	E
density	0.53	0.54			
sdensity	0.09				
py	1.63	4.02			
poverty	0.76	1.44	poverty	0.87	1.4
asian	− 0.10	− 0.51			
sjanjul	− 0.62		sjanjul	− 1.32	− 4.16

Where the variables are as defined in Table 2

## Analysis of residuals

**Fig. 7:**
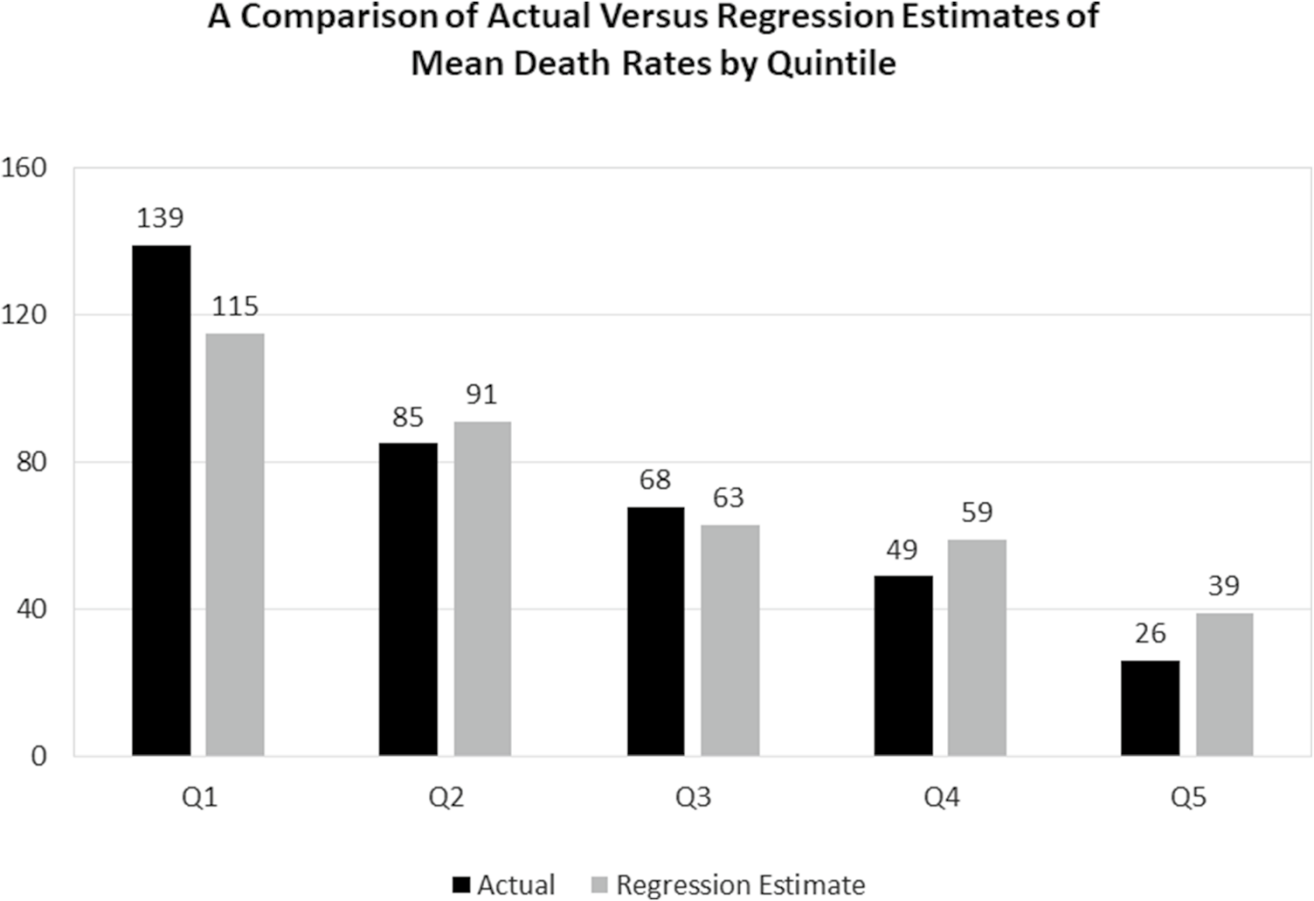
A comparison of actual versus regression estimates of mean death rates by quintile

The actual and fitted (regression estimates) and residuals for Table 3, Regression 6 during the 1/1/2020 to 12/1/2020 period are presented in Table 11. The high degree of explanatory power of the equation is shown in Fig. 7 that compares quartiles of the actual mean death rates with the corresponding mean fitted rates arranged from the highest to the lowest actual quintile values.

**Table 11:**
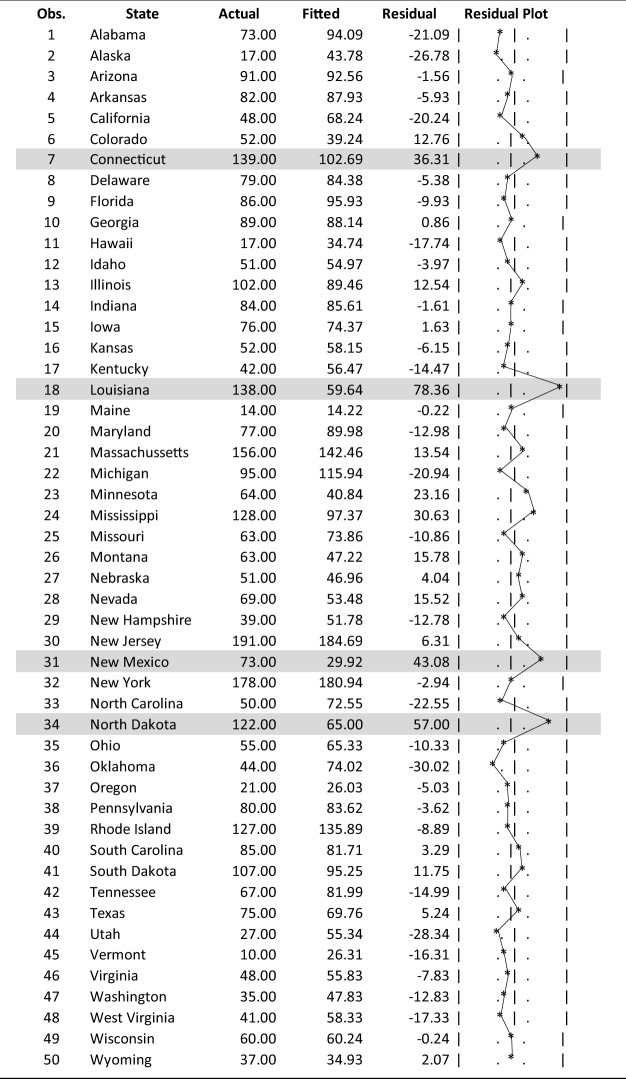
Actual mean death rates versus regression mean estimates

Quintile 1 (Q1) in Fig. 7 shows that the mean fitted value of 115 was significantly lower than the mean actual value of 139 for those states experiencing the highest 20 percent of actual death rates. In contrast to this, Fig. 7 also shows that for Quintile 5 (Q5), the mean fitted value of 39 was significantly greater than the actual value of 26 for the states having the lowest death rates. These empirical results suggest that the explanatory variables included in the best-fit Regression 6 in Table 3 do not capture the extreme values at either end of the death rate quintile range. That result, in turn, suggests that there are other factors at work other than the explanatory variables tested in this study that may help explain the high and low death rates at either end of the death rate range.

The states whose estimated death rates deviated more than ± 1.5 standard errors (± 26.4) from the actual values, as highlighted in Table 11, are shown in Table 12.

**Table 12:** Estimated COVID-19 death rates deviating more than 1.5 standard errors (± 33) from the actual values

State	Actual	Fitted	Residual
Connecticut	139	102.7	36.3
Louisiana	138	59.6	78.4
New Mexico	73	29.9	43.3
North Dakota	122	65.0	57.0

One might question why the actual death rates in the states shown in Table 12 deviated so sharply from the regression estimates. Although a rigorous examination of question is beyond the scope of this study, several observations are in order.

The fact that Connecticut experienced higher unexplained COVID-19 deaths is almost certainly due to a large percentage of its populations commuting to New York (*Hartford Current*, May 1, 2020). While our regression equations were able to capture New York’s high death rate as a result of adding a variable that measured its extraordinarily high density (sdensity), that variable was not relevant for contiguous states that were closely connected to New York City’s urban core. This argument is supported by examining the residuals for Connecticut during the 1/1/2020 to 7/1/2020 and 7/1/2020 to 12/1/2020 periods. The residuals for Connecticut only occurred during the first half of the year when density was the most significant variable explaining death rates. When density ceased to be significant in the second half of the year, the residual for Connecticut was close to zero.

In the case of Louisiana, many have suggested that its high COVID-19 death rate is due to its relatively high share of African Americans who disproportionately suffered from the coronavirus (*The Advocate*, April 24, 2020) as well as the state’s higher incidence of diabetes and obesity. This model, however, held these factors constant in our regression tests. In light of this, we believe it is more likely that the higher transmission during the early stages of the coronavirus was due to its celebration of Mardi Gras in late February. Following the celebration, Louisiana experienced the fastest growth in COVID-19 infection rates in the world (Katy Reckdahl et al., *New York Times*, updated April 13, 2020). An analysis of the residuals during the first and second half of the year supports this argument. The most extreme residual occurred during the first half of the year when the Mardi Gras occurred.

In the case of New Mexico, its actual death rates in both parts of the year were greater than one standard error and greater than the fitted values. This finding suggests that whatever led to New Mexico’s high unexplained death rate was present throughout 2020. There are many anecdotal accounts that attribute New Mexico’s high COVID-19 death rate to the large percentage of Native Americans in the population (*Albuquerque Journal*, May 30, 2020). But that percentage of 10.7% is lower than the proportion of the Native American population of 13.2% in Oklahoma. Yet, Oklahoma’s actual COVID-19′s death rate is lower than that predicted by the regression equations during all three periods tested in this study. It is also argued that Native American’s exposure to uranium extracted from Navajo lands as well as heavy metal exposure from poor air quality have weakened Native American’s immune systems (*Albuquerque Journal*, 2020). Further study outside the scope of this study is warranted to investigate these theories and others that might account for the unexplained variation in New Mexico’s high COVID-19 death rate.

The high unexplained death rate in North Dakota may be explained by the 10-day Sturgis Motorcycle Rally that is attended by almost half a million people in early August. Although this Rally is held in Sturgis, South Dakota, some believe it played a role in the surging caseload and high death rate in North Dakota.

Dr. Robert Gutter, an emergency physician at Lennox Hill Hospital, stated,

The Sturgis Motorcycle Rally was a factor in the spread of the virus throughout the Dakotas, but also the U.S., with cases reported in 11 additional states. (Healthline, 2020)

Mark Walker and Jack Healy of the New York Times described the 10-day Rally as “a Woodstock of unmasked, uninhibited coronavirus defiance” (*New York Times*, December 6, 2020, p. 6).

**Fig. 8:**
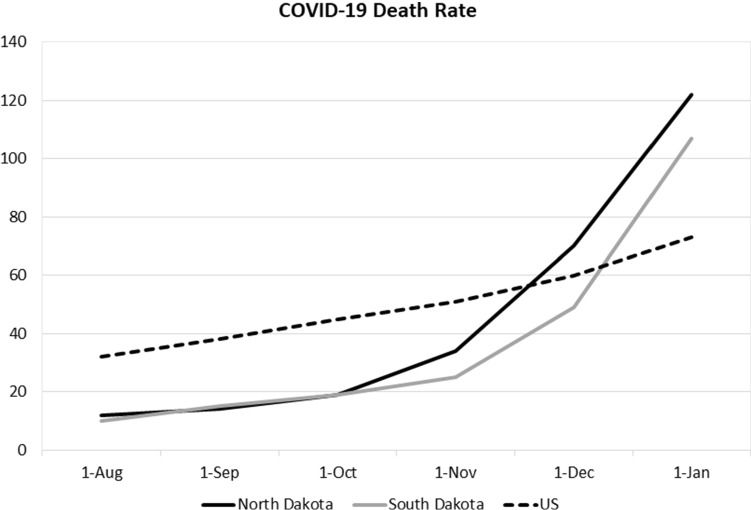
COVID-19 death rate

The evidence supports Dr. Gatter’s view. During the 1/1/2020 to 7/1/2020 regression tests, both South and North Dakota had relatively low death rates, and the regression’s fitted values were close to the actuals. The wide deviations occurred during the second half of the year. As shown in Fig. 8, the death rates in North and South Dakota after the Sturgis Rally in early August followed a similar trajectory.

Just as the Mardi Gras in New Orleans led to a spike in Louisiana’s COVID-19 death rate during the first half of the year, it appears the Sturgis Rally had similar effects in spreading the coronavirus during the second half of the year.

Analyzing the residuals from the regression equations in this study makes it possible to identify the circumstances that may be state-specific. That analysis is only possible after holding macro-oriented explanatory factors at the state level constant.

## Conclusion

A great deal of attention has been given to the actions taken by state governments and their governors to control the spread of COVID-19 and reduce its death toll. These actions have engendered much controversy over their efficacy. In spite of this, no academic papers have been published that examine and explain statewide differences in COVID-19 infections and deaths. This study hopes to fill that gap by presenting a step-wise regression model that measures the impact of hypothesized explanatory variables on each state’s COVID-19 death rate.

An important finding in this study with critically important policy ramifications is that the density of a state’s population is the most important factor explaining a state’s death rate during the first half of the year, but its impact on COVID-19 death rates during the second half registered no significance. This finding runs counter to several studies that found that population is more important than density in explaining infections and deaths from the coronavirus. The fact that this study reached different conclusions is not because it is more macro in scope. More likely, it stems from different research and methodological designs. In addition, the declining significance of density during the course of 2020 points to the critical importance of measuring the impact of the causal variables on COVID-19 during different periods of time.

The empirical findings in this study also suggest that higher poverty rates are significantly associated with higher COVID-19 death rates. This finding is supported during all periods tested in this study. Specifically, the elasticity calculations suggest that a 1% increase in a state’s poverty rate leads on average to a 0.76 increase during the 1/1/2020 to 7/1/2020 period and a 0.87% increase during the 7/1/2020 to 12/1/2020 period.

Race and ethnicity were found to have no significant impact on COVID-19 deaths. The only exception to this is a finding that a greater percentage of Asian Americans (asians) reduced a state’s COVID-19 death rate during the early stages of the virus.

The step-wise regression methodology used in this study made it possible to measure the impact of governmental mandates on COVID-19 death rates while holding other significant explanatory factors constant. Measuring the stringency of those mandates was made possible by the Oxford index that measured the stringency of 11 different mandates on a daily basis. Although mandates were found to reduce COVID-19 death rates during the first half of the year, the findings in this study suggest that their efficacy increased over time. The elasticities measured in this study, for example, suggest that a one percent increase in the Oxford index led to a reduction of 0.62% in COVID-19 deaths during the 1/1/2020 to 7/1/2020 period as compared to a reduction of 1.32% during the 7/1/2020 to 12/1/2020 period. The estimated coefficients reported in Tables 4 and 5 also made it possible to estimate the impact of a state’s mandate strategy on the level of COVID-19 deaths.

An examination of the residuals from our best-fit equation suggests that the widest differences between estimated and actual death rates are mainly due to unique circumstances in various states. The fact that our model identified those states opens interesting lines of future research.

Future research should also be directed at tracking and updating the regression findings in this study as more data become available and to testing whether the methodology and model structure in this study are applicable at the county and MSA levels. The empirical results of this study should also be considered in developing more effective strategies in the distribution of COVID-19 vaccines.
